# Treatment discontinuation rates due to lack of efficacy through 1 year of maintenance treatment with vedolizumab or subcutaneous infliximab in patients with inflammatory bowel disease: a systematic literature review and meta-analysis

**DOI:** 10.1177/17562848251383767

**Published:** 2025-10-08

**Authors:** Marc Ferrante, Laurent Peyrin-Biroulet, Perttu Arkkila, Alessandro Armuzzi, Jean-Frédéric Colombel, Silvio Danese, Roberto Faggiani, Jordi Guardiola, Stephen B. Hanauer, Jorgen Jahnsen, Walter Reinisch, Xavier Roblin, Philip J. Smith, Taek Sang Kwon, Seungmin Kim, Kyoungwan Nam, Raja Atreya

**Affiliations:** Department of Gastroenterology and Hepatology, University Hospitals Leuven, Leuven, Belgium; Department of Chronic Diseases and Metabolism, KU Leuven, Leuven, Belgium; Department of Gastroenterology, INFINY Institute, FHU-CURE, INSERM NGERE, Nancy University Hospital, Vandœuvre-lès-Nancy, France; Groupe Hospitalier Privé Ambroise Paré–Hartmann, Neuilly-sur-Seine, France; Division of Gastroenterology and Hepatology, McGill University Health Centre, Montreal, Quebec, Canada; Department of Gastroenterology, Helsinki University, Helsinki, Finland; Helsinki University Hospital, Helsinki, Finland; IBD Center, IRCCS Humanitas Research Hospital, Rozzano, Milan, Italy; Department of Biomedical Sciences, Humanitas University, Pieve Emanuele, Milan, Italy; Icahn School of Medicine at Mount Sinai, New York, NY, USA; Gastroenterology and Endoscopy, University Vita-Salute San Raffaele, Milan, Italy; Gastroenterology Department, San Camillo Hospital, Rome, Italy; Digestive Diseases Department, Bellvitge University Hospital, Bellvitge Biomedical Research Institute-IDIBELL, University of Barcelona, L’Hospitalet de Llobregat, Barcelona, Spain; Division of Gastroenterology and Hepatology, Department of Medicine, Feinberg School of Medicine, Northwestern University, Chicago, IL, USA; Institute of Clinical Medicine/Department of Gastroenterology, University of Oslo/Akershus University Hospital, Oslo, Norway; Department of Internal Medicine III, Medical University of Vienna, Vienna, Austria; University Hospital of Saint-Etienne, Saint-Etienne, France; Honorary Consultant Gastroenterologist, Department of Gastroenterology, Royal Liverpool Hospital, Liverpool University Hospitals NHS Foundation Trust, Liverpool, UK; Celltrion, Inc., Incheon, Republic of Korea; Celltrion, Inc., Incheon, Republic of Korea; Celltrion, Inc., Incheon, Republic of Korea; Medical Department 1, University Hospital Erlangen, Friedrich-Alexander-University of Erlangen-Nürnberg, Ulmenweg 18, 91054 Erlangen, Germany

**Keywords:** alpha 4 beta 7 integrin inhibitor, Crohn’s disease, treatment discontinuation, tumor necrosis factor inhibitor, ulcerative colitis

## Abstract

**Background:**

Infliximab (IFX) and vedolizumab (VDZ), frequently used biologics in inflammatory bowel disease (IBD), are available as intravenous (IV) and subcutaneous (SC) formulations; however, comparative data are limited.

**Objectives:**

To compare the rates of discontinuation due to lack of efficacy during maintenance treatment with infliximab (subcutaneous) and vedolizumab (intravenous and subcutaneous) in patients with moderate-to-severe IBD.

**Design:**

Systematic literature review and meta-analysis.

**Data sources and methods:**

Three medical databases, PubMed, Embase, and the Cochrane Library, were systematically searched from January 2010 to May 2024 to identify phases I–III randomized controlled trials. The primary outcome was discontinuation of study drug due to lack of efficacy (per definitions used in the included studies) during maintenance treatment (PROSPERO number CRD42023438330). Rates of discontinuation due to adverse events during maintenance treatment were examined in additional exploratory analyses.

**Results:**

We identified three eligible clinical trials in IBD for subcutaneous infliximab (591 patients) and five for vedolizumab (intravenous and subcutaneous formulations; 2117 patients). Rates of discontinuation due to lack of efficacy (per individual study definition) were significantly lower in patients treated with subcutaneous infliximab (0.05 (95% confidence interval (CI): 0.03, 0.06)) than in patients treated with vedolizumab (0.29 (95% CI: 0.20, 0.38)); rates remained significantly lower with subcutaneous infliximab versus vedolizumab, respectively, in the subgroups of patients with Crohn’s disease (0.05 (95% CI: 0.02, 0.07) vs 0.37 (95% CI: 0.27, 0.47)) or ulcerative colitis (0.05 (95% CI: 0.02, 0.07) vs 0.24 (95% CI: 0.11, 0.36)). Rates of discontinuation due to adverse events were lower in subcutaneous infliximab-treated patients (0.04 (95% CI: 0.02, 0.05) than in vedolizumab-treated patients (0.08 (95% CI: 0.05, 0.11)).

**Conclusion:**

In this meta-analysis, rates of discontinuation due to lack of efficacy during maintenance treatment were lower with subcutaneous infliximab than with vedolizumab (intravenous and subcutaneous formulations) in patients with moderate-to-severe IBD.

**Trial registration:**

PROSPERO number CRD42023438330.

## Introduction

The therapeutic landscape for inflammatory bowel disease (IBD) has been transformed with the introduction of biologic agents and new small-molecule therapies. Biologics targeting tumor necrosis factor (TNF), including infliximab (IFX) and adalimumab, have achieved a fundamental role in the management of IBD, and potential treatment options have expanded with the availability of newer biologics, including the integrin alpha4beta7 blocker vedolizumab (VDZ) and IL-12/23 inhibitor ustekinumab, and small-molecule Janus kinase inhibitors such as upadacitinib.

Both IFX and VDZ are recommended as first-line biologic treatments for patients with moderately to severely active Crohn’s disease (CD) or ulcerative colitis (UC) with an inadequate response or intolerance to conventional therapy.^1,2^ Both agents are initially administered via intravenous (IV) infusions at 0, 2, and 6 weeks to induce clinical remission (i.e., induction treatment), and are subsequently administered either intravenously or subcutaneously at fixed intervals to maintain clinical remission (i.e., maintenance treatment).^1–4^ Given that these two agents share similar indications for use but have distinct mechanisms of action, direct comparisons of their relative efficacy and safety can assist clinicians in optimizing treatment decisions tailored to individual patient needs, ultimately improving long-term clinical outcomes.

When selecting any treatment, evidence will help inform the choice. However, only one head-to-head clinical trial has so far been conducted comparing TNFis versus anti-integrin agents in patients with IBD: the VARSITY study compared the TNFi adalimumab with VDZ in patients with moderate-to-severe active UC and found VDZ to be superior to adalimumab for achieving clinical remission and endoscopic improvement, but not corticosteroid-free clinical remission.^5^ With a lack of head-to-head clinical trial data for IFX and VDZ in patients with IBD, systematic review and meta-analysis of data from randomized controlled trials can provide indirect evidence on the comparative efficacy or safety of these commonly used treatment options. As such, network meta-analyses can provide important information that may guide clinical practice in IBD.^6–9^

Treatment selection is further complicated by the availability of both IV and subcutaneous (SC) formulations in Europe and North America.^3,4,10^ The availability of SC formulations offers patients greater flexibility and may increase the uptake of these agents. With the recent availability of IFX SC in Europe^4^ and North America,^10^ it is important to conduct an evidence synthesis for the SC formulation of IFX compared with vedolizumab. Following the publication of data from the phase III LIBERTY studies of IFX SC in patients with moderately to severely active CD (LIBERTY-CD) and UC (LIBERTY-UC),^11^ we have conducted a systematic literature review and meta-analysis to compare rates of discontinuation due to lack of efficacy during maintenance treatment with IFX SC and VDZ (IV and SC) in patients with moderate-to-severe IBD. Discontinuation due to lack of efficacy is a relevant, patient-centered outcome that reflects both clinical effectiveness and long-term treatment durability and may provide additional insights into therapeutic performance.

## Materials and methods

This systematic literature review and meta-analysis was conducted according to a prospectively registered study protocol (PROSPERO number CRD42023438330).

### Search strategy

We conducted systematic electronic searches of PubMed, Embase, and the Cochrane Library using the search terms shown in Supplemental Tables 1 and 2. In addition, reference lists from relevant systematic reviews were cross-checked versus the electronic search results. Searches were restricted to English-language publications reporting human studies published between January 1, 2010, and May 22, 2024.

### Criteria for the consideration of studies to be included in the analysis

#### Study design

Phases I–III randomized (placebo- or active-) controlled trials were eligible for consideration. Studies performed in single countries involving small numbers of patients were excluded. Protocols (without results), case reports/studies, notes, commentaries, letters, editorials, opinions, and economic model studies were not included.

#### Participants

Data from adults (aged ⩾18 years) with moderately to severely active CD or UC were analyzed.

#### Interventions

Data were obtained from trials comparing IFX SC with VDZ (IV or SC).

#### Outcome measures

The primary prespecified outcome of interest was discontinuation of study drug due to lack of efficacy during maintenance treatment, per definitions used in the included studies. Discontinuation of study drug due to adverse events during maintenance treatment was evaluated as an additional exploratory outcome.

### Evidence synthesis and quality assessment

#### Study selection

Two authors (S.K. and K.N.) independently screened the titles and abstracts of the retrieved records to identify studies as either potentially relevant for inclusion or as irrelevant to the research question and to be excluded (noting reasons). Full-text publications for potentially relevant studies were then sourced and reviewed independently by two authors (S.K. and K.N.) to determine inclusion/exclusion. The inclusion or exclusion of the literature was ultimately determined through discussion with a third author (T.K.).

**Figure 1.**
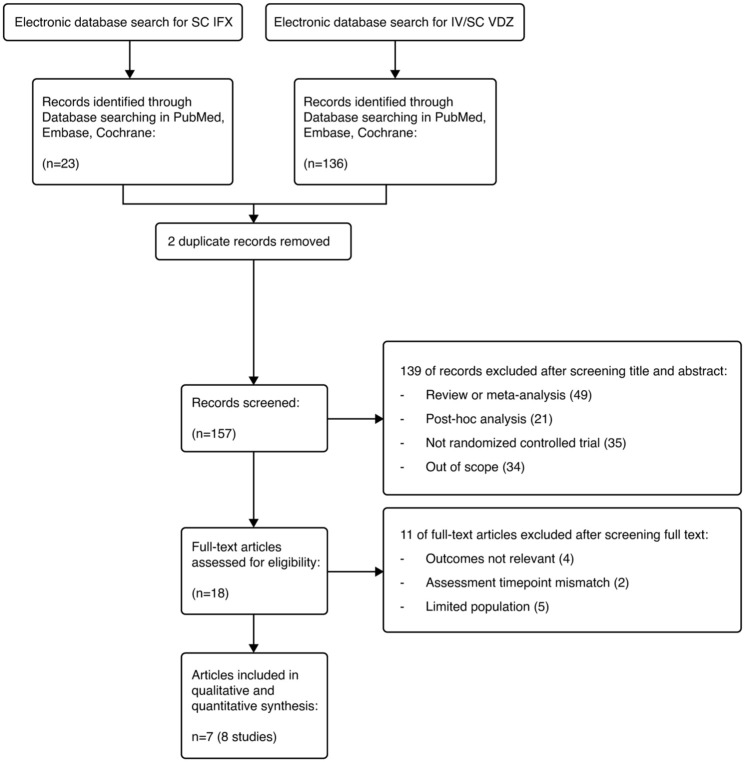
PRISMA flow diagram. IFX, infliximab; PRISMA, Preferred Reporting Items for Systematic Reviews and Meta-Analyses; SC, subcutaneous; VDZ, vedolizumab.

The study selection process was documented using a Preferred Reporting Items for Systematic Reviews and Meta-Analyses (PRISMA) flow chart (Figure 1).^12^

#### Data extraction

Study characteristics and outcome data were extracted from the included studies and recorded using a Microsoft Excel template. The extracted data included the following: design (study duration and maintenance regimen), population (disease type, total enrollment, numbers of patients receiving maintenance treatment), interventions, and prespecified outcome measure (see above).

#### Quality assessment

Risk of bias for the included studies was assessed independently by two authors (S.K. and K.N.) according to criteria outlined in the Cochrane Handbook for Systematic Reviews of Interventions,^13^ using Review Manager (RevMan version 5.4.1; The Cochrane Collaboration). The risk of bias was evaluated via seven domains: random sequence generation (selection bias); allocation concealment (selection bias); blinding of participants and personnel (performance bias); blinding of outcome assessment (selection bias); incomplete outcome data (attrition bias); selective reporting (reporting bias); and other bias. For each potential source of bias, the risk was rated as low, high, or unclear.

### Statistical methods

Data from multiple studies were pooled using a two-sided random-effect meta-analysis. Pooled rates (with 95% confidence intervals (CI)) of discontinuation due to lack of efficacy (primary outcome of interest) and discontinuation due to adverse events (exploratory outcome) were calculated using a random effects model and reported as forest plots. Effect sizes and 95% CIs were compared between IFX SC and VDZ (IV and SC) in the overall population (i.e., CD + UC) and subgroups of patients with CD or UC. Significance was defined as a *p*-value <0.05. Heterogeneity across trials was estimated using the *I*^2^ statistic.

To examine the impact of differences in study design across the included studies, sensitivity analyses were conducted to evaluate the effect of excluding non-responders at week 6, VDZ formulation (IV and SC), and the previous biologic history (experienced vs naïve) of patients treated with IFX SC.

Statistical analyses were performed using MetaProp in R (version 4.2.2; R Foundation, Vienna, Austria).

## Results

### Search results and qualitative description of the included studies

A PRISMA flow diagram summarizing the flow of information in this systematic review is presented in Figure 1. The initial searches identified a total of 159 records (23 for IFX SC and 136 for VDZ (IV and SC)). After removal of 2 duplicates, 157 records were screened (139 records were excluded), and 18 full-text articles were assessed for eligibility. Eleven articles were subsequently excluded due to irrelevant outcomes, a mismatch in assessment time points, and a limited population, including three records relating to studies or subanalyses conducted in single countries, with small sample sizes.^14–16^ Finally, seven articles (eight studies) were included in the qualitative and quantitative syntheses.

Two relevant study articles were identified for IFX SC: CT-P13 SC 1.6 study (NCT02883452),^17^ LIBERTY-CD (NCT03945019), and LIBERTY-UC (NCT04205643).^11^ Five relevant study articles were identified for VDZ (IV and SC): GEMINI 2 (NCT00783692),^18^ VISIBLE 2 (NCT02611817),^19^ GEMINI 1 (NCT00783718),^20^ VARSITY (NCT02497469),^5^ and VISIBLE 1 (NCT02611830).^21^

The key characteristics of the studies, including participant information, are presented in Supplemental Table 3. All the studies were randomized, multicenter, international clinical trials with a duration of 54 weeks (for the three studies evaluating IFX SC) or 52 weeks (for the five studies evaluating VDZ). In all the studies involving patients with CD, participants were required to be aged ⩾18 years with a Crohn’s Disease Activity Index (CDAI) score of 220–450 points and inadequate response or intolerance to previous treatment. For the studies involving patients with UC, participants were required to be aged ⩾18 years with a Mayo score of 5–12 and an endoscopic subscore of ⩾2 after having received previous treatment (e.g., corticosteroids, azathioprine, mercaptopurine, TNFi).

Of the eight studies, seven included double-blind assessment of IFX or VDZ maintenance therapy; in the CT-P13 SC 1.6 study, patients received open-label maintenance treatment with IFX. In six of the eight studies, IFX or VDZ maintenance therapy was compared with placebo; VARSITY involved comparison of VDZ IV maintenance treatment with adalimumab, and the CT-P13 SC 1.6 study was a parallel-group study with cohorts receiving maintenance treatment with IFX SC or IFX IV. In VISIBLE 1, patients received maintenance therapy with VDZ SC, VDZ IV, or placebo, while patients in VISIBLE 2 received maintenance therapy with VDZ SC or placebo. In four of the five studies involving VDZ (GEMINI 1, GEMINI 2, VISIBLE 1, and VISIBLE 2) and two of the three studies involving IFX (LIBERTY CD and LIBERTY UC), there was selective inclusion of induction responders into the maintenance phase.

A summary of the treatment regimens for each study is provided in Supplemental Table 3. Across the VDZ studies, maintenance treatment regimens with VDZ were 300 mg IV Q4W, 300 mg IV Q8W, or 108 mg SC Q2W. Across the IFX studies, the maintenance treatment regimen with IFX SC was 120 mg Q2W; however, in the CT-P13 SC 1.6 study, dosing was weight-based, whereby patients with body weight <80 kg received CT-P13 SC 120 mg Q2W and patients with body weight ⩾80 kg received CT-P13 SC 240 mg Q2W. In the LIBERTY studies, dose adjustment was permitted from weeks 22 through 102, whereby patients receiving either CT-P13 SC 120 mg Q2W or placebo SC Q2W could receive a dose adjustment to CT-P13 SC 240 mg Q2W if they initially achieved a response but subsequently met the criteria for loss of response (for CD, defined as an increase in CDAI score of ⩾100 from week 10 with a total CDAI score of ⩾220; for UC, defined as an increase in modified Mayo score ⩾2 points and ⩾30% from the week 10 modified Mayo score with an actual value of ⩾5 points, and an endoscopic subscore of ⩾2 points).^11^

The quantitative analyses included data from a total of 591 patients receiving IFX SC and 2117 patients receiving VDZ. Across the treatment arms in all studies, mean/median age ranged from 32.3 to 41.6 years, and 38.3%–53.4% of participants were female. In studies with available data, mean disease duration ranged from 5.7 to 9.5 years (Supplemental Table 3).

### Risk of bias

A summary of the risk of bias assessment is presented in Supplemental Figure 1. Across 56 assessments (considering the 8 studies and the 7 risk-of-bias domains), 48 were considered at low risk of bias and 8 at high risk. All eight studies had a low risk of bias with regard to random sequence generation, allocation concealment, blinding of outcome assessment, incomplete outcome data, and selective reporting. Six studies had a high risk of “other” bias: VISIBLE 1, VISIBLE 2, LIBERTY CD, and LIBERTY UC, due to selective inclusion of induction responders only in the maintenance phase; VARSITY, due to the unexplained disparity between primary and secondary remission outcomes; and the CT-P13 SC 1.6 study, which was considered at high risk of bias with regard to blinding of participants due to the open-label study design.

### Discontinuation due to lack of efficacy

**Figure 2.**
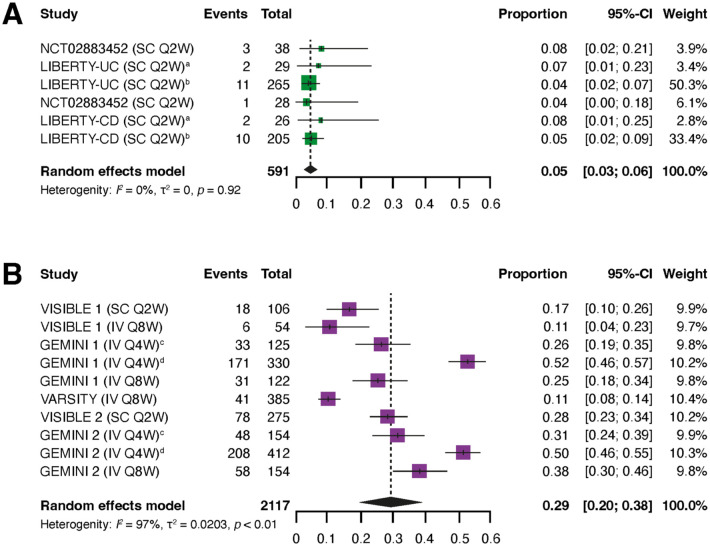
Pooled rates of discontinuation due to lack of efficacy in patients with IBD who received maintenance treatment with IFX SC (a) or VDZ (IV or SC) (b). ^a^Patients with previous experience of biologic therapy. ^b^Patients without previous experience of biologic therapy. ^c^Patients achieving a response after 6 weeks of induction treatment. ^d^Patients not achieving a response after 6 weeks of induction treatment. CD, Crohn’s disease; CI, confidence interval; IBD, inflammatory bowel disease; IFX, infliximab; IV, intravenous; Q#W, every # weeks; SC, subcutaneous; UC, ulcerative colitis; VDZ, vedolizumab.

During the maintenance phase, rates of discontinuation due to lack of efficacy were significantly lower in patients treated with IFX SC than in patients treated with VDZ (IV or SC). In the overall population of patients with IBD, the pooled rate of discontinuation due to lack of efficacy was 0.05 (95% CI: 0.03, 0.06) with IFX SC and 0.29 (95% CI: 0.20, 0.38) with VDZ (IV or SC; Figure 2 and Supplemental Table 4). Heterogeneity (*I*^2^) was markedly greater for the VDZ (IV or SC) studies compared with the IFX SC studies.

**Figure 3.**
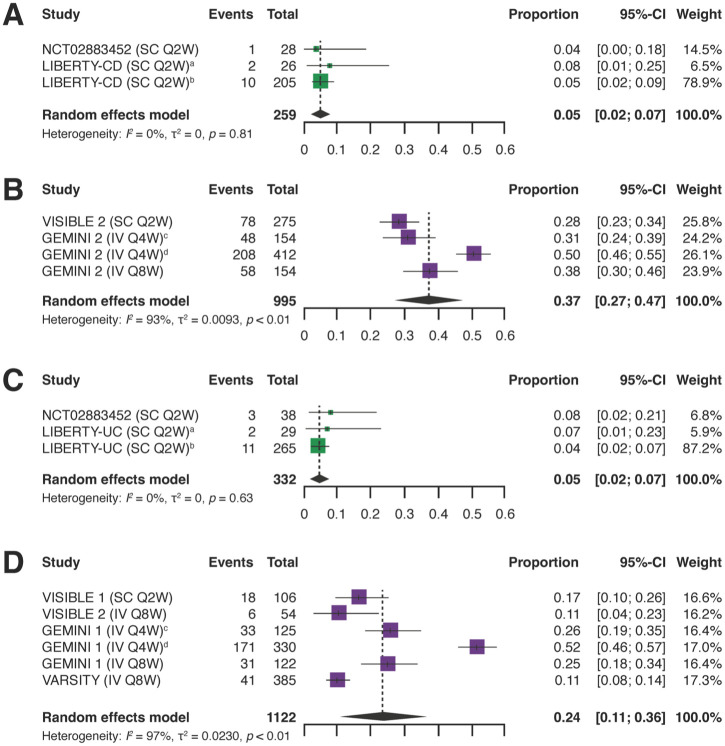
Pooled rates of discontinuation due to lack of efficacy in patients with CD who received maintenance treatment with IFX SC (a) or VDZ (IV and SC) (b) and in patients with UC who received maintenance treatment with IFX SC (c) or VDZ (IV or SC) (d). ^a^Patients with previous experience of biologic therapy. ^b^Patients without previous experience of biologic therapy. ^c^Patients achieving a response after 6 weeks of induction treatment. ^d^Patients not achieving a response after 6 weeks of induction treatment. CD, Crohn’s disease; CI, confidence interval; IBD, inflammatory bowel disease; IFX, infliximab; IV, intravenous; Q#W, every # weeks; SC, subcutaneous; UC, ulcerative colitis; VDZ, vedolizumab.

Rates of discontinuation due to lack of efficacy were also significantly lower with IFX SC than VDZ (IV or SC) in the subgroups of patients with CD and UC. In patients with CD, the pooled rate of discontinuation due to lack of efficacy was 0.05 (95% CI: 0.02, 0.07) with IFX SC and 0.37 (95% CI: 0.27, 0.47) with VDZ (IV or SC; Figure 3 and Supplemental Table 4). In patients with UC, corresponding values were 0.05 (95% CI: 0.02, 0.07) with IFX SC and 0.24 (95% CI: 0.11, 0.36) with VDZ (IV or SC; Figure 3 and Supplemental Table 4). Similar to the findings for the overall IBD population, heterogeneity (*I*^2^) was markedly greater for the VDZ (IV or SC) studies compared with the IFX SC studies, both in patients with CD or UC.

#### Sensitivity analyses

In sensitivity analyses, exclusion of non-responders to 6 weeks of VDZ induction therapy in the GEMINI 1 and GEMINI 2 studies led to slightly lower rates of discontinuation due to lack of efficacy for VDZ (Supplemental Figure 2 and Supplemental Table 4); however, the difference between IFX SC and VDZ (IV or SC) remained statistically significant in the overall IBD population and CD/UC subgroups. In additional sensitivity analyses evaluating the VDZ formulation (IV or SC) or previous biologic history (experienced vs naïve) of patients treated with IFX SC, rates of discontinuation due to lack of efficacy were similar between VDZ IV or SC groups, and biologic-experienced versus biologic-naïve groups in IFX SC-treated patients (Supplemental Figures 3 and 4, and Supplemental Table 4). With regard to formulation, rates of discontinuation due to lack of efficacy were significantly lower with IFX SC than with VDZ SC (Supplemental Figure 3 and Supplemental Table 4).

#### Discontinuation due to adverse events

Exploratory analyses showed that, during the maintenance phase, rates of discontinuation due to adverse events were numerically lower in patients treated with IFX SC than in patients treated with VDZ (IV or SC). In the overall population of patients with IBD, the pooled rate of discontinuation due to adverse events was 0.04 (95% CI: 0.02, 0.05) with IFX SC and 0.07 (95% CI: 0.04, 0.10) with VDZ (IV or SC; Supplemental Figure 5). Rates of discontinuation due to adverse events were numerically lower in CD and similar in UC with IFX SC compared with VDZ (IV or SC) in the subgroups of patients with CD and UC. In patients with CD, the pooled rate of discontinuation due to adverse events was 0.04 (95% CI: 0.02, 0.06) with IFX SC and 0.09 (95% CI: 0.03, 0.14) with VDZ (IV or SC; Supplemental Figure 6). In patients with UC, corresponding values were 0.04 (95% CI: 0.02, 0.05) with IFX SC and 0.06 (95% CI: 0.03, 0.09) with VDZ (IV or SC; Supplemental Figure 7).

## Discussion

The present study compared rates of discontinuation due to lack of efficacy during maintenance treatment with IFX SC and VDZ (IV and SC) in patients with moderate-to-severe IBD. Our study demonstrated that rates of discontinuation due to lack of efficacy were significantly lower with IFX SC compared with VDZ in an overall population of patients with IBD, as well as subgroups of patients with CD or UC during maintenance treatment. Findings were consistent after exclusion of data for non-responders to VDZ induction therapy. Sensitivity analyses of VDZ formulation (SC or IV) and previous biologic history of IFX SC-treated participants (biologic-experienced vs biologic-naïve) showed limited effect on the rates of discontinuation due to lack of efficacy. Finally, in exploratory analyses, pooled rates of discontinuation due to adverse events were numerically lower with IFX SC than VDZ (SC or IV) in the overall population of patients with IBD and in the subgroups of patients with CD and UC. IBD clinical studies often include worsening disease as an adverse event. Differences in such adverse events between IFX SC and VDZ (SC or IV) may relate to a lower risk of disease flares during maintenance treatment with IFX relative to VDZ. The observed difference in discontinuation rates between IFX SC and VDZ (24% absolute risk reduction and number needed to treat to prevent one discontinuation = 4.16 for IFX SC vs VDZ) has clinical implications, potentially leading to improved treatment persistence, better long-term patient outcomes, and reduced treatment-switching costs.^22^

The risk of bias associated with studies included in this meta-analysis was generally considered to be low. Some aspects of the studies were, however, associated with a high risk of bias: VISIBLE 1, VISIBLE 2, LIBERTY CD, and LIBERTY UC only included induction responders in the maintenance phase; VARSITY reported inconsistent results for the primary and secondary outcomes relating to clinical remission; and the CT-P13 SC 1.6 study was an open-label trial. Inclusion of only responders in the maintenance treatment phase has the potential to overestimate the efficacy or safety of the maintenance therapy, which could affect the results of the present analysis by reducing discontinuations due to lack of efficacy. Indeed, sensitivity analyses showed that excluding non-responders did lead to slightly lower rates of discontinuation due to lack of efficacy for VDZ, but the difference between IFX SC and VDZ remained statistically significant. Therefore, the findings of this analysis are most applicable to induction responders, and additional real-world studies are needed to confirm their relevance in unselected patient populations. Open-label designs can influence both patient and investigator expectations, potentially leading to improved adherence or reporting bias.

The present meta-analysis is the first to specifically evaluate IFX SC. A previously performed indirect comparison of data from IFX (IV and SC formulations) and VDZ randomized controlled trials in adults with moderate-to-severe IBD showed that, compared with VDZ, IFX had better efficacy in the induction phase, comparable efficacy in the maintenance phase, and a similar overall safety profile.^23^

Reports of persistence rates with IFX and VDZ have been published,^24–26^ as discussed below; however, caution should be exercised in interpreting the published results in the context of our current meta-analysis, as factors beyond discontinuations due to lack of efficacy will influence reported rates (e.g., discontinuations due to adverse events, as examined in exploratory analyses herein). Two retrospective, observational studies based on commercial US claims databases reported similar unadjusted persistence rates with IV IFX and VDZ in biologic-naïve individuals with CD (IFX, 79.0% of 1127 treated patients; VDZ, 78.9% of 342), biologic-experienced individuals with CD (IFX, 77.4% of 341; VDZ, 80.8% of 593),^24^ and in biologic-naïve individuals with UC (IFX, 72.5% of 1885; VDZ, 78.5% of 873) and biologic-experienced individuals with UC (IFX, 70.2% of 524; VDZ, 78.1% of 1019).^25^ Although the latter study was not intended to compare biologic-naïve and biologic-experienced treatment groups,^25^ the results between these groups were similar. This finding is consistent with those of the subanalyses in the present study, which showed similar rates of discontinuation due to lack of efficacy in biologic-naïve and biologic-experienced patients who received CT-P13 SC as maintenance treatment (noting that subgroup analysis by biologic treatment history was not possible for VDZ based on the included studies). A limitation of the aforementioned observational studies is that some data (including reasons for discontinuation) could not be obtained, as claims databases are designed for reimbursement purposes.^25^ In another retrospective, observational study, involving biologic-naïve patients with moderate-to-severe UC, drug persistence rates of 52% and 78% with IFX and VDZ, respectively, were reported.^26^ However, it is important to note that this was a single-center study with a small sample size: only 82 individuals (50 receiving IFX and 32 receiving VDZ) were evaluated in this analysis.^26^ In a real-world study of US electronic medical records from biologic-naïve patients with IBD receiving IFX (*n* = 1179) or VDZ (*n* = 542), persistence rates were 77.5% and 84.5%, respectively, at 12 months and 64.6% and 77.6%, respectively, at 24 months.^27^ However, reasons for discontinuation were not reported, and caution should be exercised when comparing real-life data with results from our meta-analysis, which is based on controlled data from randomized clinical trials. Differences in study design, such as observational studies versus randomized trials, can affect outcomes due to variations in patient populations, treatment settings, and confounding factors.

Strengths of the current analysis include it being the first study to evaluate drug persistence in patients receiving IFX SC versus VDZ (IV or SC) and the use of validated methodology (e.g., comprehensive electronic searches; independent screening of search results by two authors; assessment of risk of bias).^13^ Weaknesses of our study include that the analysis was limited to IFX and VDZ and did not consider other guideline-recommended treatment options for moderate-to-severe IBD (e.g., tofacitinib and ustekinumab); the analyzed population included both biologic-experienced and biologic-naïve individuals; disparities in sample size among the included studies may have influenced the meta-analysis weighting and potentially introduced bias into the pooled effect estimates; and the comparison was made between IFX SC and both IV and SC formulations of VDZ (noting that only one study reporting data for VDZ SC was available for each indication—VISIBLE1 for UC and VISIBLE2 for CD—therefore, a pooled analysis of data by indication could not be conducted). We performed sensitivity analyses to account for limitations arising from VDZ IV/SC data and biologic history; however, there was little available published data for VDZ SC, and the sample sizes for the sensitivity analyses were small. The lack of data available from the clinical studies made potentially informative subgroup analyses difficult to perform; for example, the sensitivity analysis of previous biologics was restricted to IFX-treated patients due to data availability. In addition, treatment optimization strategies, such as tailored dosing frequency and intensification of IFX,^28^ were not utilized in the clinical trials, and real-life experience may reflect more favorable results for IFX. The high level of statistical heterogeneity in the VDZ studies indicates substantial variation between studies, which may affect the precision of the pooled estimates. In addition, the lack of stratification by prior biologic exposure may introduce confounding and should be considered when interpreting the results. Future research should include head-to-head clinical trials, long-term safety data, and analyses of different patient subgroups.

Accepting the limitations of the study, participant demographics of the study populations contributing to the analyses were representative of patients with moderately to severely active IBD and support the generalizability of the findings from the current analysis. The available results show the benefits of IFX SC in patients with IBD, and support use as a first-line biologic agent of choice in IBD, given the reduced rate of discontinuation due to lack of efficacy and comparative efficacy and safety data available.^23^

## Conclusion

In conclusion, rates of discontinuation due to lack of efficacy during maintenance treatment were lower with IFX SC than with VDZ (IV and SC) in patients with moderately to severely active IBD.

## Supplemental Material

sj-docx-1-tag-10.1177_17562848251383767 – Supplemental material for Treatment discontinuation rates due to lack of efficacy through 1 year of maintenance treatment with vedolizumab or subcutaneous infliximab in patients with inflammatory bowel disease: a systematic literature review and meta-analysis Supplemental material, sj-docx-1-tag-10.1177_17562848251383767 for Treatment discontinuation rates due to lack of efficacy through 1 year of maintenance treatment with vedolizumab or subcutaneous infliximab in patients with inflammatory bowel disease: a systematic literature review and meta-analysis by Marc Ferrante, Laurent Peyrin-Biroulet, Perttu Arkkila, Alessandro Armuzzi, Jean-Frédéric Colombel, Silvio Danese, Roberto Faggiani, Jordi Guardiola, Stephen B. Hanauer, Jorgen Jahnsen, Walter Reinisch, Xavier Roblin, Philip J. Smith, Taek Sang Kwon, Seungmin Kim, Kyoungwan Nam and Raja Atreya in Therapeutic Advances in Gastroenterology

sj-docx-2-tag-10.1177_17562848251383767 – Supplemental material for Treatment discontinuation rates due to lack of efficacy through 1 year of maintenance treatment with vedolizumab or subcutaneous infliximab in patients with inflammatory bowel disease: a systematic literature review and meta-analysis Supplemental material, sj-docx-2-tag-10.1177_17562848251383767 for Treatment discontinuation rates due to lack of efficacy through 1 year of maintenance treatment with vedolizumab or subcutaneous infliximab in patients with inflammatory bowel disease: a systematic literature review and meta-analysis by Marc Ferrante, Laurent Peyrin-Biroulet, Perttu Arkkila, Alessandro Armuzzi, Jean-Frédéric Colombel, Silvio Danese, Roberto Faggiani, Jordi Guardiola, Stephen B. Hanauer, Jorgen Jahnsen, Walter Reinisch, Xavier Roblin, Philip J. Smith, Taek Sang Kwon, Seungmin Kim, Kyoungwan Nam and Raja Atreya in Therapeutic Advances in Gastroenterology

## Declarations

**Ethics approval and consent to participate:** Not applicable.

**Consent for publication:** Not applicable.

**Competing interests:** M.F. reports research support from AbbVie, EG, Janssen, Pfizer, Takeda, and Viatris; speaker’s fees from AbbVie, Biogen, Boehringer Ingelheim, Falk, Ferring, Janssen-Cilag, MSD, Pfizer, Takeda, Truvion Healthcare, and Viatris; and consultancy fees from AbbVie, Agomab Therapeutics, Boehringer Ingelheim, Celgene, Celltrion, Eli Lilly, Janssen-Cilag, MRM Health, MSD, Pfizer, Takeda, and Thermo Fisher. L.P.-B. reports consulting and/or lecture fees from: AbbVie, Abivax, Adacyte, Alimentiv, Alma Bio Therapeutics, Amgen, Applied Molecular Transport, Arena, Biogen, BMS, Celltrion, CONNECT Biopharm, Cytoki Pharma, Enthera, Ferring, Fresenius Kabi, Galapagos, Genentech, Gilead, Gossamer Bio, GSK, HAC-Pharma, IAG Image Analysis, Index Pharmaceuticals, Inotrem, Janssen, Lilly, Medac, Mopac, Morphic, MSD, Norgine, Nordic Pharma, Novartis, OM Pharma, ONO Pharma, OSE Immunotherapeutics, Pandion Therapeutics, Par’Immune, Pfizer, Prometheus, Protagonist, Roche, Roivant, Samsung, Sanofi, Sandoz, Takeda, Theravance, Thermo Fisher, TiGenix, Tillots, VectivBio, Ventyx, Viatris, Vifor, and YSOPIA. A.A. reports consulting/advisory board fees from AbbVie, Alfa-Sigma, Amgen, AstraZeneca, Biogen, Boehringer Ingelheim, Bristol-Myers Squibb, Celltrion, Eli-Lilly, Ferring, Galapagos, Gilead, Giuliani, Janssen, Lion Health, MSD, Nestlé, Pfizer, Protagonist Therapeutics, Roche, Sanofi, Samsung Bioepis, Sandoz, Takeda, and Tillots Pharma; speaker’s fees from AbbVie, AG Pharma, Amgen, Biogen, Bristol-Myers Squibb, Celltrion, Eli Lilly, Ferring, Galapagos, Gilead, Janssen, Lion Health, MSD, Novartis, Pfizer, Roche, Samsung Bioepis, Sandoz, Takeda, and Teva Pharmaceuticals; and research grants from Biogen, MSD, Pfizer, and Takeda. P.J.S. has received educational speaker fees from AbbVie, Amgen, BMS, Celltrion, Dr Falk Pharmaceuticals, Eli Lilly, Fresenius Kabi, Galapagos, Janssen-Cilag, Takeda, and Tillotts Pharma. He has been an advisory board member for AbbVie, BMS, Celltrion, Galapagos, and Janssen-Cilag. T.K., S.K., and K.N. are employees of Celltrion Inc. R.A. has served as a speaker, consultant, or received research grants or travel support from AbbVie, Abivax, AstraZeneca, Bristol-Myers Squibb, Celltrion Healthcare, Falk Foundation, Galapagos, Janssen-Cilag, Lilly, MSD Sharp & Dohme, Roche Pharma, and Takeda Pharma. P.A., J.-F.C., S.D., R.F., J.G., S.B.H., J.J., W.R., and X.R. have nothing to declare.

**Availability of data and materials:** Data for the included analyses are presented in the main manuscript and Supplemental Materials.
