# Supplementary material for: Treatment discontinuation rates due to lack of efficacy through 1 year of maintenance treatment with vedolizumab or subcutaneous infliximab in patients with inflammatory bowel disease: a systematic literature review and meta-analysis
Source: Ther Adv Gastroenterol. 2025 Oct 8;18:17562848251383767. doi: 10.1177/17562848251383767 (PMC12511697; doi:10.1177/17562848251383767)
Supplement: sj-docx-1-tag-10.1177_17562848251383767 – Supplemental material for Treatment discontinuation rates due to lack of efficacy through 1 year of maintenance treatment with vedolizumab or subcutaneous infliximab in patients with inflammatory bowel disease: a systematic literature review and meta-analysi [file sj-docx-1-tag-10.1177_17562848251383767.docx]

Treatment Discontinuation Rates Due to Lack of Efficacy Through 1 Year of Maintenance Treatment With Vedolizumab or Subcutaneous Infliximab in Patients With Inflammatory Bowel Disease: A Systematic Literature Review and Meta-Analysis

# Supplementary Tables

**Supplementary Table 1.** Search terms (subcutaneous infliximab; PubMed).

| Search | Query |
| --- | --- |
| #1 | "Crohn's disease"[Title/Abstract] OR "Crohns disease"[Title/Abstract] OR "crohn*"[Title/Abstract] OR "CD"[Title/Abstract] OR "ulcerative colitis"[Title/Abstract] OR "ulcerative"[Title/Abstract] OR "UC"[Title/Abstract] OR "IBD"[Title/Abstract] OR "inflammatory bowel disease"[Title/Abstract] OR "Crohn Disease"[MeSH Terms] OR "colitis, ulcerative"[MeSH Terms] |
| #2 | (Infan*[Title/Abstract] OR newborn*[Title/Abstract] OR new-born*[Title/Abstract] OR perinat*[Title/Abstract] OR neonat*[Title/Abstract] OR baby[Title/Abstract] OR baby*[Title/Abstract] OR babies[Title/Abstract] OR toddler*[Title/Abstract] OR minors[Title/Abstract] OR minors*[Title/Abstract] OR boy[Title/Abstract] OR boys[Title/Abstract] OR boyfriend[Title/Abstract] OR boyhood[Title/Abstract] OR girl*[Title/Abstract] OR kid[Title/Abstract] OR kids[Title/Abstract] OR child[Title/Abstract] OR child*[Title/Abstract] OR children*[Title/Abstract]) OR adolescen*[Title/Abstract] OR juvenil*[Title/Abstract] OR youth*[Title/Abstract] OR teen*[Title/Abstract] OR under*age*[Title/Abstract] OR (pediatric*[Title/Abstract] OR paediatric*[Title/Abstract] OR peadiatric*[Title/Abstract] OR prematur*[Title/Abstract] OR preterm*[Title/Abstract]) OR (pediatrics[Mesh]) |
| #3 | "subcutaneous infliximab"[Title] OR "infliximab subcutaneous"[Title] OR "SC infliximab"[Title] OR "infliximab SC"[Title] OR "IFX subcutaneous"[Title] OR "subcutaneous IFX"[Title] OR "IFX SC"[Title] OR "SC IFX"[Title] OR "CT-P13"[Title] |
| #4 | "randomized controlled trial"[Publication Type] OR "controlled clinical trial"[Publication Type] OR ("randomised"[Title/Abstract] OR "randomized"[Title/Abstract]) OR "placebo"[Title/Abstract] OR "randomly"[Title/Abstract] OR "trial"[Title/Abstract]) NOT ("animals"[MeSH Terms] NOT "humans"[MeSH Terms] |
| #5 | #1 NOT #2 |
| #6 | #5 AND #3 |
| #7 | #6 AND #4 |

**Supplementary Table 2.** Search terms (vedolizumab; PubMed).

| Search | Query |
| --- | --- |
| #1 | "Crohn's disease"[Title/Abstract] OR "Crohns disease"[Title/Abstract] OR "crohn*"[Title/Abstract] OR "CD"[Title/Abstract] OR "ulcerative colitis"[Title/Abstract] OR "ulcerative"[Title/Abstract] OR "UC"[Title/Abstract] OR "IBD"[Title/Abstract] OR "inflammatory bowel disease"[Title/Abstract] OR "Crohn Disease"[MeSH Terms] OR "colitis, ulcerative"[MeSH Terms] |
| #2 | (Infan*[Title/Abstract] OR newborn*[Title/Abstract] OR new-born*[Title/Abstract] OR perinat*[Title/Abstract] OR neonat*[Title/Abstract] OR baby[Title/Abstract] OR baby*[Title/Abstract] OR babies[Title/Abstract] OR toddler*[Title/Abstract] OR minors[Title/Abstract] OR minors*[Title/Abstract] OR boy[Title/Abstract] OR boys[Title/Abstract] OR boyfriend[Title/Abstract] OR boyhood[Title/Abstract] OR girl*[Title/Abstract] OR kid[Title/Abstract] OR kids[Title/Abstract] OR child[Title/Abstract] OR child*[Title/Abstract] OR children*[Title/Abstract]) OR adolescen*[Title/Abstract] OR juvenil*[Title/Abstract] OR youth*[Title/Abstract] OR teen*[Title/Abstract] OR under*age*[Title/Abstract] OR (pediatric*[Title/Abstract] OR paediatric*[Title/Abstract] OR peadiatric*[Title/Abstract] OR prematur*[Title/Abstract] OR preterm*[Title/Abstract]) OR (pediatrics[Mesh]) |
| #3 | "vedolizumab"[Title] OR "Entyvio"[Title] OR "MLN0002"[Title] OR "MLN02"[Title] OR "LDP-02"[Title] |
| #5 | "randomized controlled trial"[Publication Type] OR "controlled clinical trial"[Publication Type] OR ("randomised"[Title/Abstract] OR "randomized"[Title/Abstract]) OR "placebo"[Title/Abstract] OR "randomly"[Title/Abstract] OR "trial"[Title/Abstract]) NOT ("animals"[MeSH Terms] NOT "humans"[MeSH Terms] |
| #6 | #1 NOT #2 |
| #7 | #6 AND #3 |
| #8 | #7 AND #5 |

**Supplementary Table 3.** Characteristics of the included studies.

| Study Name | Disease | Study Duration, Weeks | Maintenance Regimen | N, Total  Enrollment | N, Maintenance Period | Age, Median (Range), Years | Sex, Female, % | Disease Duration, Mean (SD), Years |
| --- | --- | --- | --- | --- | --- | --- | --- | --- |
| IFX SC | | | | | | | | |
| CT-P13 1.6 | CD/UC | 54 | CT-P13 SC 120/240 mg Q2W^a^  CT-P13 IV 5 mg/kg Q8W | Induction: 136 Maintenance: 131^b^ | CT-P13 SC: 66^c^ CT-P13 IV: 65^d^ | 33.0 (18–69) 36.0 (18–70) | 45.5 46.2 | 5.70 (6.01)  5.85 (6.29) |
| LIBERTY-CD | CD | 54 | CT-P13 SC 120 mg Q2W  Placebo SC 120 mg Q2W | Induction: 396 Maintenance: 343 | CT-P13 SC: 231 Placebo: 112 | 36.0 (18–75) 32.3 (18–66) | 42.0 38.4 | NC |
| LIBERTY-UC | UC | 54 | CT-P13 SC 120 mg Q2W  Placebo SC 120 mg Q2W | Induction: 548 Maintenance: 438 | CT-P13 SC: 294 Placebo: 144 | 37.0 (18–73) 39.0 (18–75) | 44.6 42.4 | NC |
| VDZ | | | | | | | | |
| GEMINI 2 | CD | 52 | VDZ IV 300 mg Q4W VDZ IV 300 mg Q8W Placebo IV | Induction: 1,115 Maintenance: 461 | VDZ: 308 Placebo: 153 | 35.7 (11.9)^e^ 38.6 (13.2))^e^ | 53.4 53.4 | 9.2 (7.8) 8.2 (7.8) |
| VISIBLE 2 | CD | 52 | VDZ SC 108 mg Q2W Placebo SC Q2W | Induction: 644 Maintenance: 410 | VDZ: 275 Placebo: 135 | 38.2 (13.9)^e^ 36.1 (12.9)^e^ | 42.9 50.7 | 9.5 (8.3) 8.2 (8.4) |
| GEMINI 1 | UC | 52 | VDZ IV 300 mg Q4W VDZ IV 300 mg Q8W Placebo IV | Induction: 895 Maintenance: 373 | VDZ: 247 Placebo: 126 | 40.1 (13.2)^e^ 41.2 (12.5)^e^ | 42.0 38.3 | 6.8 (6.2) 7.1 (7.2) |
| VARSITY | UC | 52 | VDZ IV 300 mg Q8W + placebo SC Q2W ADA 40 mg Q2W weeks + placebo IV Q8W | Total: 771 | VDZ: 385 ADA: 386 | 40.8 (13.7)^e^ 40.5 (13.4)^e^ | 39.2 44.0 | 7.3 (7.2) 6.4 (6.0) |
| VISIBLE 1 | UC | 52 | VDZ SC 108 mg Q2W + Placebo IV Q8W VDZ IV 300mg Q8W + Placebo SC Q2W Placebo SC Q2W + Placebo IV Q8W | Induction: 383 Maintenance: 216 | VDZ SC: 106 VDZ IV: 54 Placebo: 56 | 38.1 (13.1)^e^ 41.6 (14.1)^e^ 39.4 (11.7)^e^ | 38.7 42.6 39.3 | 8.0 (6.2) 8.2 (5.9) 7.4 (7.1) |

Abbreviations: ADA, adalimumab; CD, Crohn’s disease; IV, intravenous; NC, not collected; SC, subcutaneous; SD, standard deviation; UC, ulcerative colitis; VDZ, vedolizumab.

^a^Body-weight based dosing: 120 mg Q2W for <80 kg and 240 mg Q2W for ≥80 kg.
^b^CD, n=53; UC, n=78.
^c^CD, n=28; UC, n= 38.
^d^CD, n=25; UC, n=40.
^e^Mean (SD).

**Supplementary Table 4.** Discontinuation due to lack of efficacy.

| Population/Subgroup | Maintenance Treatment | N^a^ | Events^b^ | Proportion (95% CI) | Heterogeneity, *I^2^* |
| --- | --- | --- | --- | --- | --- |
| Main Analyses | | | | | |
| Overall | IFX SC | 591 | 29 | 0.05 (0.03, 0.06) | 0% |
|  | VDZ | 2117 | 692 | 0.29 (0.20, 0.38) | 97% |
| Crohn’s disease | IFX SC | 259 | 13 | 0.05 (0.02, 0.07) | 0% |
|  | VDZ | 995 | 392 | 0.37 (0.27, 0.47) | 93% |
| Ulcerative colitis | IFX SC | 332 | 16 | 0.05 (0.02, 0.07) | 0% |
|  | VDZ | 1122 | 300 | 0.24 (0.11, 0.36) | 97% |
| Sensitivity Analyses: Excluding Non-Responders to Induction Therapy | | | | | |
| Overall | IFX SC | 591 | 29 | 0.05 (0.03, 0.06) | 0% |
|  | VDZ | 1375 | 313 | 0.23 (0.17, 0.30) | 92% |
| Crohn’s disease | IFX SC | 259 | 13 | 0.05 (0.02, 0.07) | 0% |
|  | VDZ | 583 | 184 | 0.32 (0.27, 0.37) | 48% |
| Ulcerative colitis | IFX SC | 332 | 16 | 0.05 (0.02, 0.07) | 0% |
|  | VDZ | 792 | 129 | 0.18 (0.11, 0.24) | 83% |
| Sensitivity Analyses: Formulation | | | | | |
| Overall | IFX SC | 591 | 29 | 0.05 (0.03, 0.06) | 0% |
| Subgroup | VDZ IV | 1736 | 596 | 0.31 (0.20, 0.42) | 98% |
|  | VDZ SC | 381 | 96 | 0.23 (0.12, 0.34) | 84% |
| Sensitivity Analyses: IFX SC Treatment by Previous Biologic History | | | | | |
| Subgroup | Experienced | 55 | 4 | 0.07 (0.00, 0.14) | 0% |
|  | Naive | 536 | 25 | 0.05 (0.03, 0.06) | 0% |

Abbreviations: CI, confidence interval; IFX, infliximab; IV, intravenous; SC, subcutaneous; VDZ, vedolizumab.

^a^N participants; ^b^n participants who discontinued due to lack of efficacy.

# Supplementary Figures

**Supplementary Figure 1.** Risk of bias for the included studies, presented by study and domain (A) and by domain only (B).

**A**


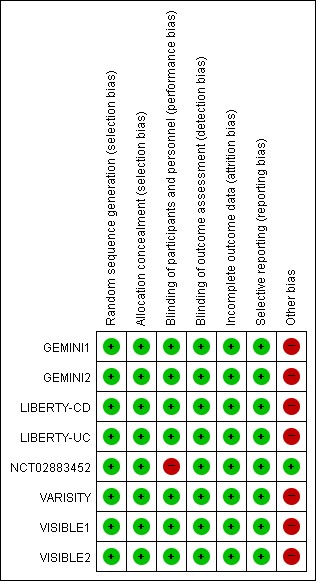


**B**

**
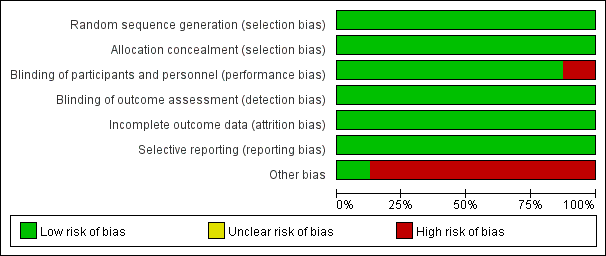
**

**Supplementary Figure 2.** Sensitivity analyses excluding non-responders to 6 weeks of VDZ induction therapy in the GEMINI 1 and 2 trials: Pooled rates of discontinuation due to lack of efficacy in responders to VDZ induction therapy in the overall group of patients with IBD (A) and in subgroups of patients with CD (B) and UC (C).

**A**

**
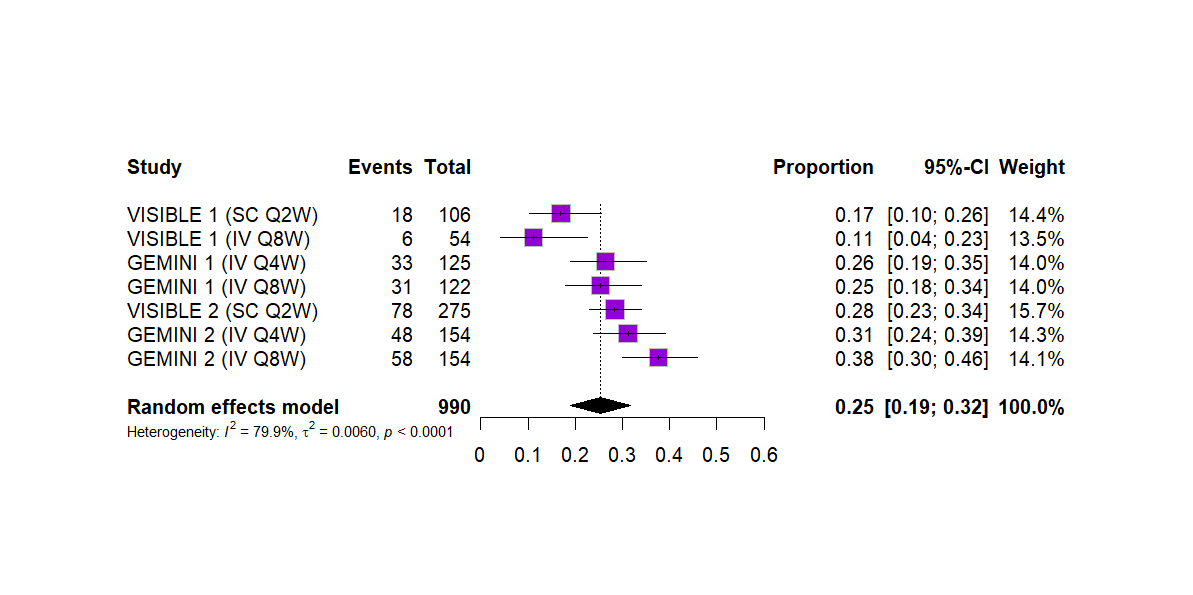
**

**B**


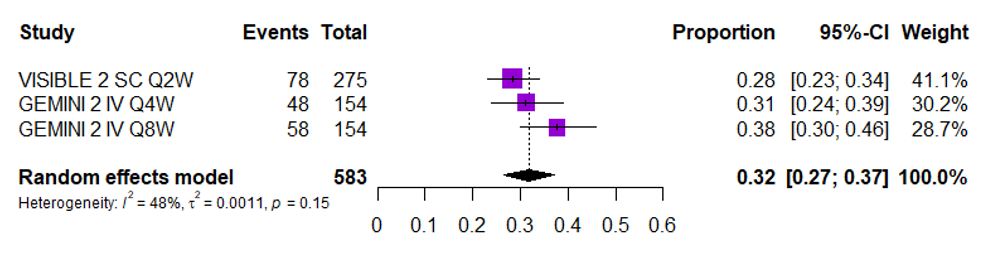


**C**


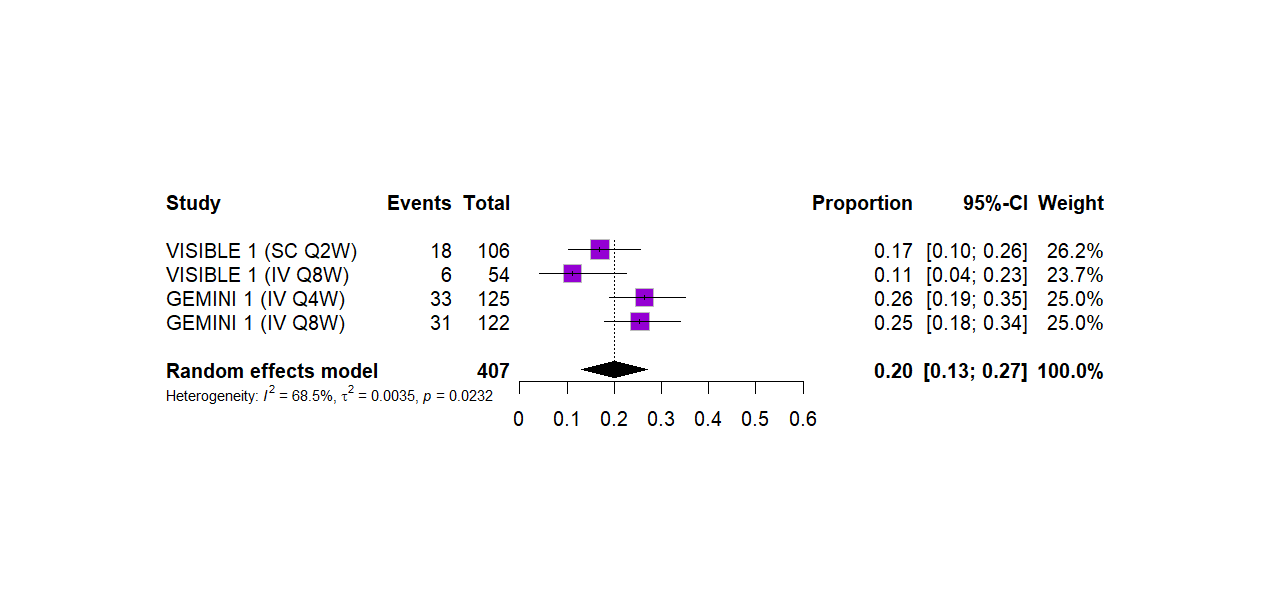


CD, Crohn’s disease; CI, confidence interval; IBD, inflammatory bowel disease; IFX, infliximab; IV, intravenous; Q#W, every # weeks; SC, subcutaneous; UC, ulcerative colitis; VDZ, vedolizumab.

**Supplementary Figure 3.** Sensitivity analyses evaluating VDZ by formulation. Pooled rates of discontinuation due to lack of efficacy in subgroups of patients receiving VDZ SC (A) and VDZ IV (B).

**A**


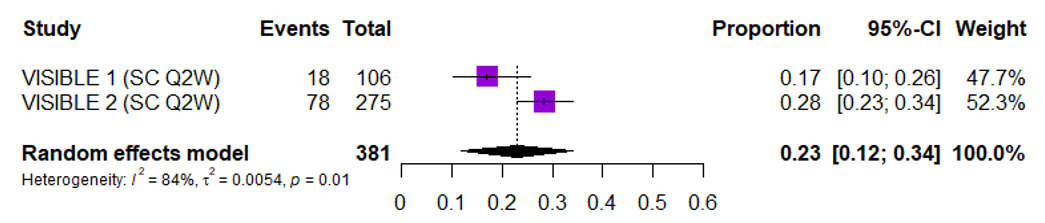


**B**


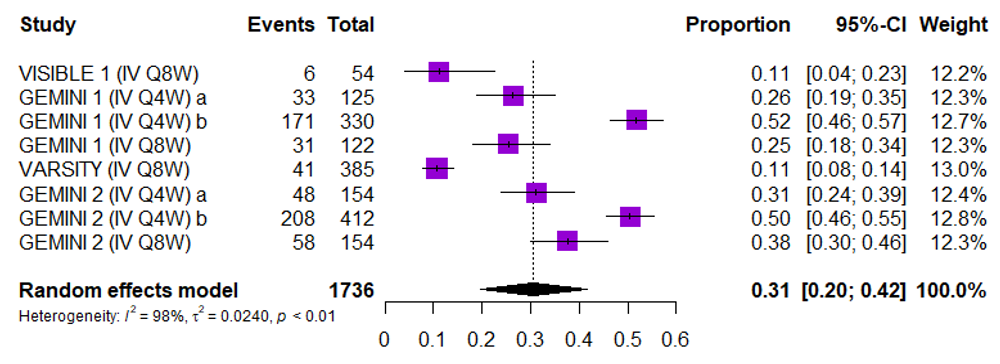


CI, confidence interval; IV, intravenous; Q#W, every # weeks; SC, subcutaneous; VDZ, vedolizumab

**Supplementary Figure 4**. Sensitivity analyses evaluating IFX SC by biologic history**.** Pooled rates of discontinuation due to lack of efficacy in subgroups of patients without previous experience of biologic therapy (A) and patients with previous experience of biologic therapy (B).

**A**


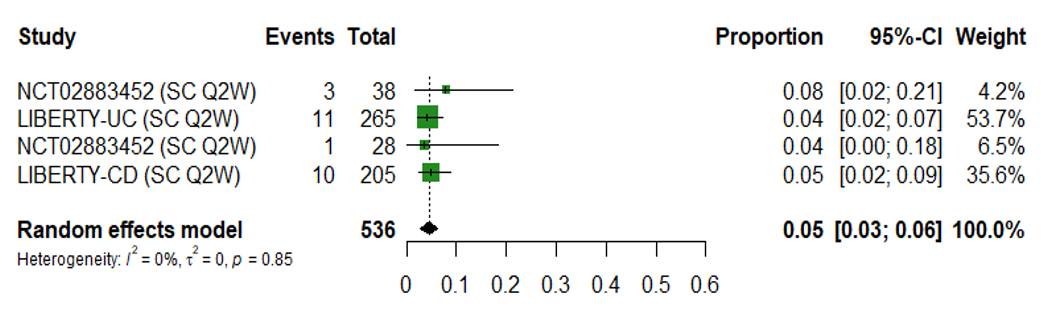


**B**


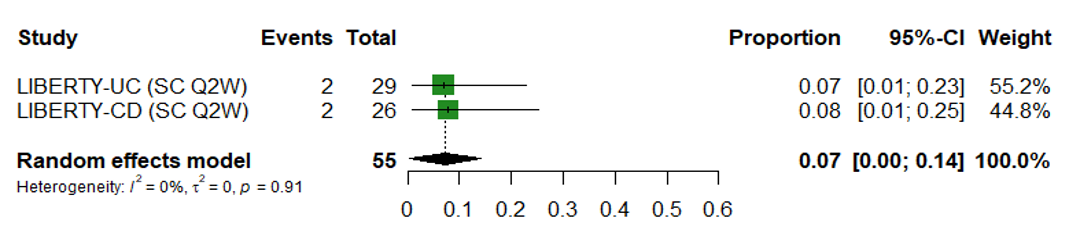


CI, confidence interval; IFX, infliximab; Q#W, every # weeks; SC, subcutaneous.

**Supplementary Figure 5.** Exploratory analysis: Pooled rates of discontinuation due to adverse events in patients with IBD who received maintenance treatment with IFX SC (A) or VDZ (IV or SC) (B).

**A**

**
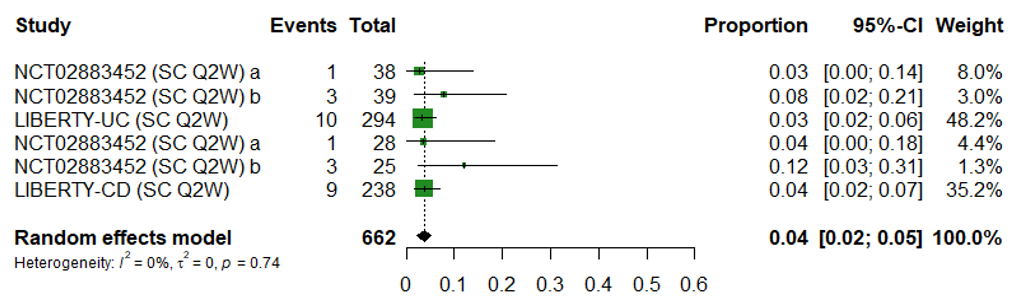
**

^a^Patients receiving IFX SC maintenance.
^b^Patients who switched from IFX IV to IFX SC.

**B**

**
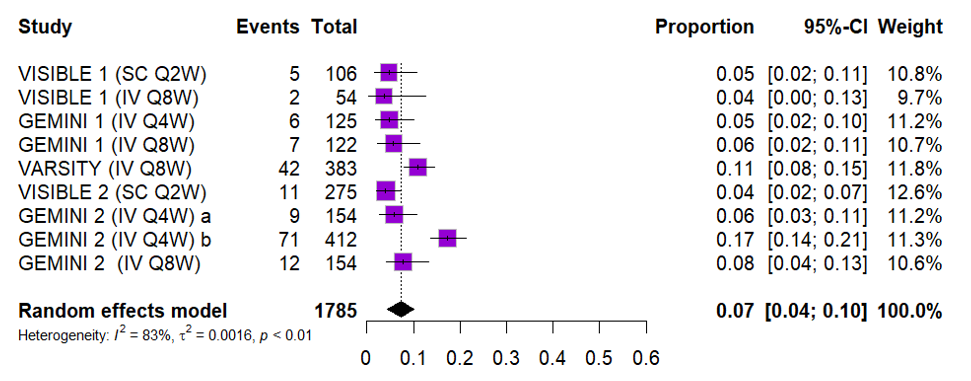
**

^a^Patients achieving a response after 6 weeks of induction treatment.
^b^Patients not achieving a response after 6 weeks of induction treatment.

CI, confidence interval; IBD, inflammatory bowel disease; IFX, inflammatory bowel disease; IV, intravenous; Q#W, every # weeks; SC, subcutaneous; VDZ, vedolizumab

**Supplementary Figure 6.** Exploratory analysis: Pooled rates of discontinuation due to adverse events in patients with CD who received maintenance treatment with IFX SC (A) or VDZ (IV and SC) (B).

**A**

**
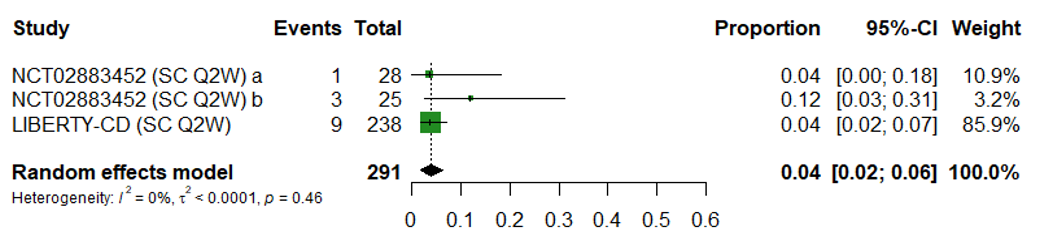
**

^a^Patients receiving IFX SC maintenance.
^b^Patients who switched from IFX IV to IFX SC.

**B**

**
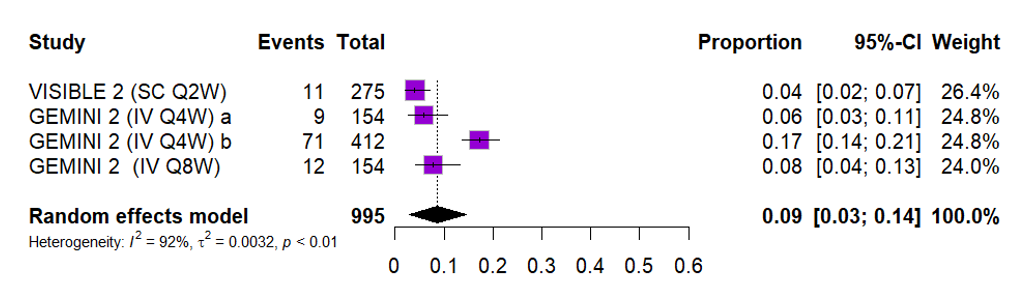
**

^a^Patients achieving a response after 6 weeks of induction treatment.
^b^Patients not achieving a response after 6 weeks of induction treatment.

CD, Crohn’s disease; CI, confidence interval; IFX, inflammatory bowel disease; IV, intravenous; Q#W, every # weeks; SC, subcutaneous; VDZ, vedolizumab

**Supplementary Figure 7.** Exploratory analysis: Pooled rates of discontinuation due to adverse events in patients with UC who received maintenance treatment with IFX SC (A) or VDZ (IV and SC) (B).

**A**

**
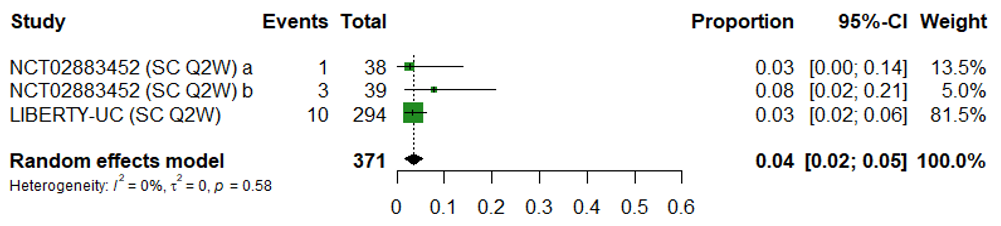
**

^a^Patients receiving IFX SC maintenance.
^b^Patients who switched from IFX IV to IFX SC.

**B**

**
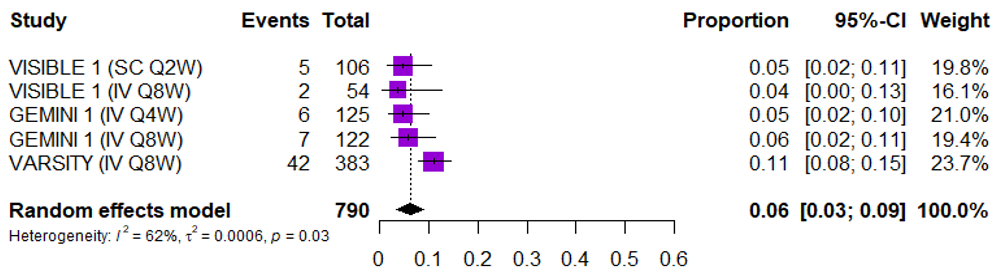
**

^a^Patients achieving a response after 6 weeks of induction treatment.
^b^Patients not achieving a response after 6 weeks of induction treatment.

CI, confidence interval; IFX, inflammatory bowel disease; IV, intravenous; Q#W, every # weeks; SC, subcutaneous; UC, ulcerative colitis; VDZ, vedolizumab.
